# Reviewed article: Oliveira GG, Gonçalves AS, Wermann H, Artus RG, Groppelli F, Gonçalves NNS. Trend in the detection rate of syphilis among older adults: time series analysis, Brazil, 2014-2023 Epidemiol Serv Saude. 2026:35;e20250900.

**DOI:** 10.1590/S2237-96222026v35e20250900.b

**Published:** 2026-04-10

**Authors:** Gabriel Pavinati

**Affiliations:** 1 Universidade Estadual de Maringá, Maringá, PR, Brazil

## Peer review B

## Questionnaire

Does the manuscript contain new and significant information to justify publication? Yes

Does the Abstract (Summary) clearly and accurately describe the content of the article? No

Is the problem significant and concisely stated? No

Are the methods described comprehensively? No

Are the interpretations and conclusions justified by the results? No

Is adequate reference made to other work in the field? Yes

## Manuscript Structure

Length of article is: Too long

Number of tables is: Adequate

Number of figures is: Too many

Please state any conflict(s) of interest that you have in relation to the review of this paper. None

Interest: Excellent

Quality: Good

Originality: Average

Overall: Good

Recommendation: Major Revision

## Comments

O estudo evidencia que a taxa de detecção de sífilis em pessoas idosas no Brasil apresentou crescimento significativo até 2020, seguido de estabilização em patamares elevados entre 2020 e 2023, totalizando mais de 117 mil notificações no período analisado. As taxas foram consistentemente mais altas no sexo masculino e alcançaram pico na faixa etária de 70 a 79 anos, com destaque para as regiões Sul e Centro-Oeste, que se mantiveram acima da média nacional. Esses achados revelam a persistência da sífilis como um problema de saúde pública nesse grupo etário e indicam disparidades de gênero e regionais que exigem atenção específica. Para o Sistema Único de Saúde, o estudo oferece evidências valiosas para o planejamento de políticas de saúde sexual voltadas às pessoas idosas, reforçando a necessidade de estratégias de prevenção, diagnóstico oportuno e cuidado continuado para mitigar os impactos da doença nessa população vulnerável.

Resumo

Na sentença “o pico de notificações ocorreu na faixa etária de 70 a 79 anos em

todas as regiões”, não ficou compreensível o que os autores afirmam, a frase está truncada. Rever.

Ainda, nesse item, carece-se de informações quantitativas sore a tendência. Qual a AAPC? Em nenhum momento foi apresentado a taxa de detecção, por exemplo.

Introdução

O termo idoso não é mais recomendado pois caracteriza o indivíduo com base em sua condição etária - o ideal é pessoa idosa - recomendo adequação na introdução e ao longo do trabalho.

Considerou-se pessoa idosa aquela acima de 65 anos, mas no Estatuto do Idoso é definido como indivíduo acima de 65 anos. Qual foi o referencial adotado pelos pesquisadores?

Na introdução são apresentados dois objetivos. Rever

Sugiro adicionar um parágrafo abordando a vulnerabilidade das pessoas idosas à sífilis, com base em estudos de fatores de risco, fundamentando e justificando o estudo.

Método

Os autores mencionam o termo `cálculo amostral`, mas quando há acesso a todos os registros do banco de dados nacional todos os casos notificados no SINAN), trata-se de um censo daquela população de interesse. Nesse caso, não se fala em cálculo de tamanho amostral, porque a análise é feita sobre o universo completo disponível. Ajustar

Informar que para os anos intercensitários foram utilizadas estimativas.

O subitem população do estudo não aborda, de fato, a população do estudo - pessoas idosas. São informados apenas sobre a coleta e a fonte de dados. Rever

Detalhar melhor como foi trabalhado o dado ignorado. Criou-se uma categoria específica para sua análise?

Para a definição geográfica do caso, considerou-se local de notificação, residência ou diagnóstico?

Houve alguma outra variável para filtrar o caso? Por exemplo, foram excluídas as notificações em que os casos tiveram teste diagnóstico negativo ou inconclusivo?

Explicitar sobre os critérios usados para a definição do número dos pontos de inflexão.

Os autores informam que pré-especificaram um único ponto de inflexão no ano de 2020. No software Joinpoint Regression Program, existe a opção direta de “pré-especificar” um ano fixo como ponto de inflexão? O que ele faz, por padrão, é testar automaticamente todos os possíveis joinpoints dentro do intervalo definido e selecionar o(s) melhor(es) pelo teste de permutação ou BIC. Qual função do programa foi utilizada?

Os autores fizeram duas analises distintas no trabalho, uma de 2014 a 2020 e outra de 2020 a 2023? Se for isso, considerando os pressupostos do Joint

Solicita-se a utilização da diretriz RECORD (Reporting of studies Conducted using Observational Routinely-collected Data) para assegurar transparência e qualidade na condução e no relato do presente estudo com dados secundários

Resultados

Os parágrafos descritivos das tabelas estão prolixos. Sugiro uma síntese mais direcionada. Alem disso, utiliza-se um linguajar pouco técnico-científico (ex: “disparam nas faixas mais velhas”). Nos resultados, são feitas inferências que extrapolam as evidências obtidas (ex: “indicando uma possível inflexão no comportamento epidemiológico da doença na região”)

A tabela 1 e a figura 1 apresentam os mesmos dados, causando redundância interpretativa. Sugiro apresentar apenas a tabela.

No método, fala-se de variáveis como: relato do início dos sintomas; faixa etária estratificada em 60 a 64, 65 a 69, 70 a 79 e 80 anos ou mais; sexo (masculino e feminino); raça/cor (branca, amarela, parda, indígena e preta); escolaridade (analfabeto, ensino fundamental, médio e superior). No entanto, elas não são apresentadas. Há incoerência. Rever.

